# Comparing clinico-demographics and neuropsychiatric symptoms for immigrant and non-immigrant aged care residents living with dementia: a retrospective cross-sectional study from an Australian dementia-specific support service

**DOI:** 10.1186/s12877-023-04447-3

**Published:** 2023-11-10

**Authors:** Pelden Chejor, Mustafa Atee, Patricia Cain, Daniel Whiting, Thomas Morris, Davina Porock

**Affiliations:** 1 Centre for Research in Aged Care, School of Nursing and Midwifery, Edith Cowan University, 270 Joondalup Drive, Joondalup, Western Australia 6027 Australia; 2 The Dementia Centre, HammondCare, Osborne Park, Western Australia Australia; 3 Curtin Medical School, Faculty of Health Sciences, Curtin University, Bentley, Western Australia Australia; 4 Sydney Pharmacy School, Faculty of Medicine and Health, The University of Sydney, Sydney, New South Wales Australia; 5 The Dementia Centre, HammondCare, St Leonards, New South Wales Australia

**Keywords:** Immigrants, Neuropsychiatric symptoms, Dementia, NPS, Non-English-speaking, Caregiver distress, Pain

## Abstract

**Background:**

Neuropsychiatric symptoms of dementia such as agitation and aggression are common in people living with dementia. The presentation of neuropsychiatric symptoms is influenced by the cultural background of people living with dementia. Further, identifying factors contributing to neuropsychiatric symptoms may be complicated if people living with dementia are immigrants or from non-English-speaking backgrounds. Most of what is known about differences in neuropsychiatric symptoms between racial and ethnic groups living with dementia come from community-based samples. This study investigated differences in clinico-demographics and neuropsychiatric symptoms between immigrants and non-immigrants living with dementia in residential aged care homes who were referred to two Dementia Support Australia programs.

**Methods:**

This was a retrospective observational cross-sectional study from 2018 to 2022 using data extracted from the Dementia Support Australia database. Immigrant status was identified by documented country of birth. We conducted exploratory subgroup analyses for English-speaking or non-English-speaking immigrants in comparison to non-immigrants. Neuropsychiatric Inventory and PainChek^®^ were used to assess neuropsychiatric symptoms of dementia and pain, respectively.

**Results:**

Of the 23,889 referrals, 36% were immigrants living with dementia. Immigrants were 0.8 years older than non-immigrants on average. Immigrants had a slightly higher prevalence of mixed dementia (9.5%) than non-immigrants (8.2%). Overall, the groups had no difference in the severity of neuropsychiatric symptoms and associated caregiver distress. However, there was a significant difference in the total number of neuropsychiatric inventory domains (Cohen’s *d* = -0.06 [-0.09, - 0.02], *p* <.001) between non-English-speaking immigrants and non-immigrants. Immigrants were more likely to present with agitation/aggression, while non-immigrants were more likely to present with hallucinations. Factors contributing to neuropsychiatric symptoms were common between the groups, with language barriers and cultural considerations frequently endorsed for immigrants.

**Conclusion:**

This study reveals a mixed picture of neuropsychiatric symptoms between immigrants and non-immigrants. However, due to the exploratory nature of the hypotheses, our findings need to be replicated in future studies to confirm any conclusions. There is a need for increased awareness on the impact of culture and language on neuropsychiatric symptoms for people receiving residential care. Future studies investigating neuropsychiatric symptoms in different immigrant groups will help increase our understanding of neuropsychiatric symptoms for all people.

**Supplementary Information:**

The online version contains supplementary material available at 10.1186/s12877-023-04447-3.

## Introduction

In Australia, over 400,000 people are living with dementia, a number projected to double by 2058 [1]. At least 54% (132,000) of people living in residential aged care homes (RACHs) in 2019 – 2020 had a dementia diagnosis [1]. Over 31% of aged care residents were also born overseas (19.6% from non-English-speaking countries and 12.2% from other English-speaking countries), and 9.2% of people using aged care preferred a language other than English [2]. In 2019 - 2020, 21% of people living with dementia in RACHs were immigrants from non-English speaking countries [1]. International studies have reported that immigrants experience a higher prevalence of dementia due to differing life experiences including those related to trauma, low literacy, and socioeconomic status [3–5].

Caring for people living with dementia presents specific challenges, such as communication due to the loss of cognitive function, loss of language, neuropsychiatric symptoms (NPS), and the complex progressive nature of the condition [6]. Care complexity can increase when the recipient is an immigrant. As dementia progresses, it is common for people from non-English-speaking backgrounds to lose their ability to communicate in English and revert to using their first language to communicate [7, 8]. Resultant communication barriers where formal care providers do not share the same language may complicate the ability of care providers to understand and meet the needs of people living with dementia [9], particularly around expression of pain and NPS.

More than 95% of people living with dementia in RACHs experience at least one NPS such as agitation or aggression [10]. However, most of what is known about differences in NPS based on racial and ethnic groups living with dementia comes from community-based samples. For people living with Alzheimer’s disease in the community, it has been reported that NPS such as hallucinations, night-time behaviour, and elation were more common for people with Hispanic backgrounds compared to people from non-Hispanic backgrounds and that the severity of NPS differed between the two groups [11]. A recent study found that NPS were more likely to occur in non-Hispanic Black *[sic]* participants with dementia than they were in non-Hispanic White *[sic]* participants with dementia [12]. Similarly, a case-control study from a Dutch urban memory clinic (case, *n* = 415, control, *n* = 428) showed that older ethnic minority patients with dementia migrating from 47 countries had significantly more depression, change in appetite, agitation/aggression, disinhibition, and aberrant motor behaviour than native Dutch people with dementia [13].

NPS can impact both people living with dementia and their caregivers. For the individual, NPS are associated with faster disease progression [14, 15] and poor quality of life [16, 17]. For caregivers, research has focused mainly on the informal caregiver burden and less on the formal caregiver perspective. NPS are related to increased informal caregiver distress [18] and burden [19, 20] as well as poor caregiver mental health [21]. Cultural diversity may also impact caregiver burden, as shown in one study, where foreign-born informal caregivers of people living with dementia reported more stress associated with NPS than native Canadian caregivers [22]. In addition, a recent systematic review found that NPS increases the burden for caregivers of older immigrants living with dementia [23]. Fauth and Gibbons [24] also noted that NPS-associated caregiver distress could be higher for family or informal caregivers than formal caregivers who are routinely dealing with NPS.

Factors contributing to the development of NPS are numerous and are often categorised as factors related to people living with dementia (e.g., neurobiologically related disease factors, medical illness, unmet needs), caregiver factors (e.g., stress, burden, communication issues), and factors related to environment or care settings (over- or under-stimulation, unsafe environment) [25]. Undiagnosed medical illnesses of people living with dementia have previously been related to NPS [26]. Pain is also strongly linked to agitation/aggression [27] as people living with dementia often express pain and discomfort by displaying aggressive behaviours [28]. Caregiver factors such as increased stress and depression were related to more NPS in care recipients [29], while changes in daily routines (e.g., bathing and dressing) were associated with stress for people living with dementia [30]. The complexity of NPS means understanding the underlying causes of NPS is important for tailoring support for people living with dementia and their caregivers. Pain, carer approach, loneliness/boredom, mood disorders, and communication difficulties were identified as the common contributing factors to NPS for people living with dementia in a national Australian sample referred to the Dementia Support Australia (DSA) programs [31]. Other factors including sleep disorders [32], sex, and education [33] were also attributed as potentially contributing to NPS. Further factors may also contribute to NPS and identifying these may be complicated in instances where people living with dementia are immigrants or from non-English-speaking backgrounds. This topic remains largely under-researched, particularly in Australia, where the older immigrant population living with dementia is projected to increase [34].

This study aimed to compare the clinico-demographic characteristics (e.g., age, sex, dementia subtype), prevalence and severity of NPS, and associated caregiver distress among people living with dementia (immigrants and non-immigrants) in RACHs referred to DSA, a national provider of dementia behaviour support. To address the objectives of this study, we set the following hypotheses:

1. there will be differences in demographic characteristics between immigrants (English-speaking or not) and non-immigrants.
2. there will be differences in the individual and overall prevalence and severity of NPS between immigrants (English-speaking or not) and non-immigrants.
3. there will be differences in overall caregiver distress reported by caregivers of immigrants (English-speaking or not) and non-immigrants.
4. there will be differences in the prevalence of contributing factors for NPS in immigrants (English-speaking or not) and non-immigrants.

## Methods

### Study design and setting

This observational cross-sectional study drew on retrospective demographic and clinical data (e.g., age, dementia subtype) from the DSA database from 1 June 2018 to 30 June 2022. DSA is a dementia-specific government-funded program in Australia that provides free-of-charge national support programs in the form of individualised psychosocial or non-pharmacological interventions for people living in various care settings with varying severity of NPS-related dementia [35]. Support from DSA includes the Dementia Behaviour Management Advisory Service (DBMAS) for mild to moderate NPS and the Severe Behaviour Response Teams (SBRT) for moderate to severe NPS. Eligibility criteria for receiving support from DSA include: (1) having a confirmed or probable diagnosis of dementia; (2) people living with dementia exhibiting NPS related to dementia; (3) people living with dementia exhibiting NPS that impacts their care and well-being, or their caregivers; and (4) receiving consent from people living with dementia or their responsible caregiver [31, 36]. This study focused on support provided by DSA in RACHs.

### Data extraction

Demographic and clinical data (age, sex, dementia subtypes, country of birth, Neuropsychiatric Inventory (NPI) scores, pain scores, and contributing factors) were extracted by a data custodian using the DSA database for RACHs referrals with NPS seeking support from DBMAS and SBRT programs [35].

### Study instruments

The NPI and PainChek^®^ are clinical instruments that DSA routinely administer to assess NPS and pain, respectively. The NPI is used during each consultation and the version of NPI used is determined by the type of DSA programs delivered. The Neuropsychiatric Inventory Questionnaire (NPI-Q) is administered for DBMAS referrals while the Neuropsychiatric Inventory Nursing Home version (NPI-NH) is administered for SBRT referrals [35].

The NPI-NH [36] and NPI-Q [37] are informant-based instruments that are valid, consistent, and reliable tools for assessing 12 NPI domains -aberrant motor behaviour, agitation/aggression, anxiety, apathy/indifference, appetite and eating, delusions, depression/dysphoria, disinhibition, elation/euphoria, hallucinations, irritability/lability, and night-time behaviour [36, 37]. The NPI-Q and NPI-NH rate each NPI domain on its presence (Yes = present, No = absent) and severity (‘mild’ (1), ‘moderate’ (2), ‘severe’ (3)) [36, 37]. The NPI-Q and NPI-NH also assess the caregiver distress/disruptiveness associated with each of the 12 NPI domains with ratings from (‘not at all’ (0), ‘minimally’ (1), ‘mildly’ (2), ‘moderately’ (3), ‘severely’ (4), ‘extremely’ (5)) [36, 37]. The total caregiver distress/disruptiveness scores can be calculated separately by adding the distress/disruptiveness scores of all 12 NPI domains. Higher scores on distress denote more distressing behaviours, while higher scores on disruptiveness denote more disruptive behaviours. For our scoring, scores from both NPI-Q and NPI-NH are referred to as total NPI scores, and the caregiver distress/disruptiveness is referred to as caregiver distress.

### Contributing factors to NPS in DSA referrals

Contributing factors to NPS are identified by DSA consultants through a comprehensive onsite assessment of referrals. This includes consultation with the referral and their formal and/or informal caregivers and a review of the referral’s clinical files such as medical records and social history. DSA consultants conduct clinical observations and interviews, collect proxy assessments from families and caregivers, and use validated instruments such as PainChek^®^ and the NPI [35]. During an assessment, DSA consultants use a comprehensive list of factors contributing to NPS and assign those applicable to the referral. The list of potential contributing factors covers several broad causes of NPS from the categories of biological, environmental, psychological, and social factors [31].

Pain is routinely assessed by DSA consultants using the PainChek^®^ Adult, a multimodal, psychometrically sound, artificial intelligence (AI)-based pain assessment tool in the form of a point-of-care app for non-verbal adults [39]. PainChek^®^ is registered as a software medical device by Australia’s Therapeutic Goods Administration, Health Canada, Singapore Health Sciences Authority, and European Conformity [38, 39]. The PainChek^®^ pain assessment scale is a 42-item instrument assessing six domains [Face (9 items), Voice (9 items), Movement (7 items), Behaviour (7 items), Activity (4 items), and Body (6 items)]. Each item is provided with a clear operational definition to improve interrater consistency, and items are rated on a binary level (Yes = present, No = absent) [38]. The final pain score ranges from 0 (no pain) to 42 (severe pain). Pain is present if the final score is ≥ 7 and absent if the final score is <7 [39, 40].

Similar to Loi et al [31], we investigated in this study the prevalence of common contributing factors of NPS plus two additional factors (language barriers and cultural considerations), which are directly related to our study objectives.

### Eligibility criteria of participants for data analysis

Data were included in the study if the data related to someone who was: (1) referred to DSA programs during the study period; (2) residing in RACH at the time of referral; and (3) aged 65 years or older. If the referrals accessed DSA services multiple times during the study period, the earliest referral was used. Referrals not meeting any of the above criteria were excluded.

### Data analysis

For analysis, data were classified into two groups according to country of birth: (1) non-immigrant group for those referrals whose country of birth was documented as Australia; (2) immigrant group for those referrals whose country of birth documented was other than Australia. The immigrant group was further classified into subgroups as English-speaking immigrants or non-English-speaking immigrants depending on whether they were born in countries where English is the primary language or not, using the World Population Review list of English-Speaking Countries 2022 [41].

We used R version 4.2.2 [42] for the statistical analyses. We considered a *p*-value of <.001 statistically significant to reduce the risk of false positives [43] as *p*-values would easily reach statistical significance in large sample sizes [44]. Demographic characteristics (age, gender, dementia subtypes) and NPS characteristics (total number of domains, severity, and caregiver distress) between groups were compared. Descriptive statistics (mean, standard deviation, frequency, and percentages) were used to describe demographic variables and assessment characteristics. Pearson’s chi-square test with Cramer’s *V* effect size was used to compare categorical demographic variables (Hypothesis 1 and 2). Cramer’s *V* effect sizes were interpreted in relation to the associated degrees of freedom of chi-square test where small effect values are in the range of 0.04 (for five degrees of freedom) to 0.10 (for 1 degree of freedom) [45].

For Hypothesis 1, 2 and 3, continuous variables were compared using Welch’s *t*-test and Cohen’s *d* effect sizes, with a 95% confidence interval. Cohen’s *d* effect sizes were interpreted as small (*d* = 0.2), medium (*d* = 0.5), and large (*d* = 0.8) [46]. A logistic regression model was used to predict immigrant status from the individual NPI domain severity scores (Hypothesis 2). Two linear regression models with immigrant status, age, and sex as predictors were also created, using total NPS severity and total caregiver distress as dependent variables, to investigate the relationship between immigrant status and total NPS severity and total caregiver distress scores. For all regression analyses, the reference group was non-immigrants living with dementia. The rates of most frequently identified contributing factors (in addition to language barriers and cultural considerations) for each group, were compared using a two-sample *Z*-test with a 95% confidence interval (Hypothesis 4).

## Results

### Descriptive characteristics

A total of 23,889 referrals to DSA were eligible for the study, of which 36% were immigrants living with dementia (*n* = 8607, 55.4% female). Table 1 shows the descriptive characteristics of non-immigrants and immigrants, with further categorisation for non-English-speaking immigrants and English-speaking immigrants. There was a significant difference in age, with immigrants being 0.8 years older than non-immigrants on average (Cohen’s *d* = 0.12 [0.09, 0.14], *p* <.001). Subgroup analyses revealed that non-English-speaking immigrants were 1.3 years older than non-immigrants on average (Cohen’s *d* = 0.18 [0.15, 0.21], *p* <.001), but without significant difference in sex. No significant differences in age and sex were observed between English-speaking immigrants and non-immigrants. There was a significant difference in the prevalence of dementia subtypes between immigrants and non-immigrants but again with small effect sizes (Cramer’s *V* = 0.03 [0.01, 0.05], *p* <.001). This effect was primarily driven by differences in mixed dementia and frontal lobe dementia, with immigrants being 1.3% higher in mixed dementia and 0.7% lower in frontal lobe dementia than non-immigrants. There were also significant differences in dementia subtypes in both subgroup comparisons, with mixed dementia being 2.2% higher in non-English speaking immigrants than non-immigrants, and Alzheimer’s disease being 4.6% higher in English-speaking immigrants compared to non-Immigrants.

**Table 1:** Descriptive characteristics of non-immigrants, immigrants, NES immigrants, and ES immigrants

**Group**	**Non-immigrants**	**Immigrants**	**Effect size [95% ** ***CI*** **]**	***p***	**NES immigrants**	**ES immigrants**
Sample size,* n* (%)	15,282 (64.0%)	8,607 (36.0%)	NA	NA	5,723 (23.9%)	2,884 (12.1%)
**Age, years**						
Mean (SD)	83.7 (7.6)	84.5 (7.0)	0.12 [ 0.09, 0.14]*	< .001	85.0 (6.9)	83.7 (7.2)
**Sex, ** ***n*** ** (%)**						
Female	8,770 (57.4%)	4,766 (55.4%)	0.02 [ 0.00, 0.03]**	.003	3,155 (55.1%)	1,611 (55.9%)
Male	6,482 (42.4%)	3,821 (44.4%)			2,554 (44.6%)	1,267 (43.9%)
Missing	18 (0.1%)	15 (0.2%)			10 (0.2%)	5 (0.2%)
Intersex or indeterminate	12 (0.1%)	5 (0.1%)			4 (0.1%)	1 (0.0%)
**Dementia subtype, ** ***n*** ** (%)**						
Alzheimer's disease	5,747 (37.6%)	3,251 (37.8%)	0.03 [ 0.01, 0.05]**	< .001	2,033 (35.5%)	1,218 (42.2%)
Dementia unspecified	4,412 (28.9%)	2,503 (29.1%)			1,734 (30.3%)	769 (26.7%)
Vascular dementia	1,910 (12.5%)	1,114 (12.9%)			767 (13.4%)	347 (12.0%)
Mixed dementia	1,260 (8.2%)	817 (9.5%)			598 (10.4%)	219 (7.6%)
Other dementias	775 (5.1%)	364 (4.2%)			232 (4.1%)	132 (4.6%)
Lewy body dementia	417 (2.7%)	220 (2.6%)			130 (2.3%)	90 (3.1%)
Frontal lobe dementia	380 (2.5%)	157 (1.8%)			107 (1.9%)	50 (1.7%)
Dementia in Parkinson's disease	238 (1.6%)	104 (1.2%)			70 (1.2%)	34 (1.2%)
Missing	143 (0.9%)	77 (0.9%)			52 (0.9%)	25 (0.9%)
**NPI Totals, Mean (SD)**						
Severity	10.8 (5.9)	10.8 (5.8)	0.00 [-0.03, 0.02]*	.746	10.6 (5.7)	11.2 (5.8)
Caregiver distress	14.6 (8.6)	14.6 (8.6)	0.00 [-0.03, 0.03]*	.923	14.3 (8.6)	15.0 (8.6)
Number of Domains	5.2 (2.2)	5.2 (2.2)	-0.02 [-0.05, 0.01]*	.269	5.1 (2.2)	5.3 (2.2)
**Pain Present, ** ***n*** ** (%)**						
PainChek® score ≥7 (Pain present)	5,039 (68.0%)	3,016 (68.4%)	0.00 [0.00, 0.02]**	.720	2,036 (69.9%)	980 (65.3%)
PainChek® score <7 (Pain absent)	2,367 (32.0%)	1,395 (31.6%)			875 (30.1%)	520 (34.7%)

Percentages for the NES and ES immigrants are calculated using the number of all immigrants

*CI, p-*value, and effect sizes are only applicable to non-immigrants and all immigrants

^*^Cohen’s *d* effect size; **Cramer’s *V* effect size, *NES* Non-English-speaking, *ES* English-speaking, *NA* Not applicable, *NPI* Neuropsychiatric inventory, *CI* Confidence interval, *p* probability value, *SD* Standard deviation, Other dementias: alcohol-related dementia, dementia in human immunodeficiency virus, dementia in Huntington’s disease and dementia in other substance abuse

### Neuropsychiatric symptoms

**Fig. 1:**
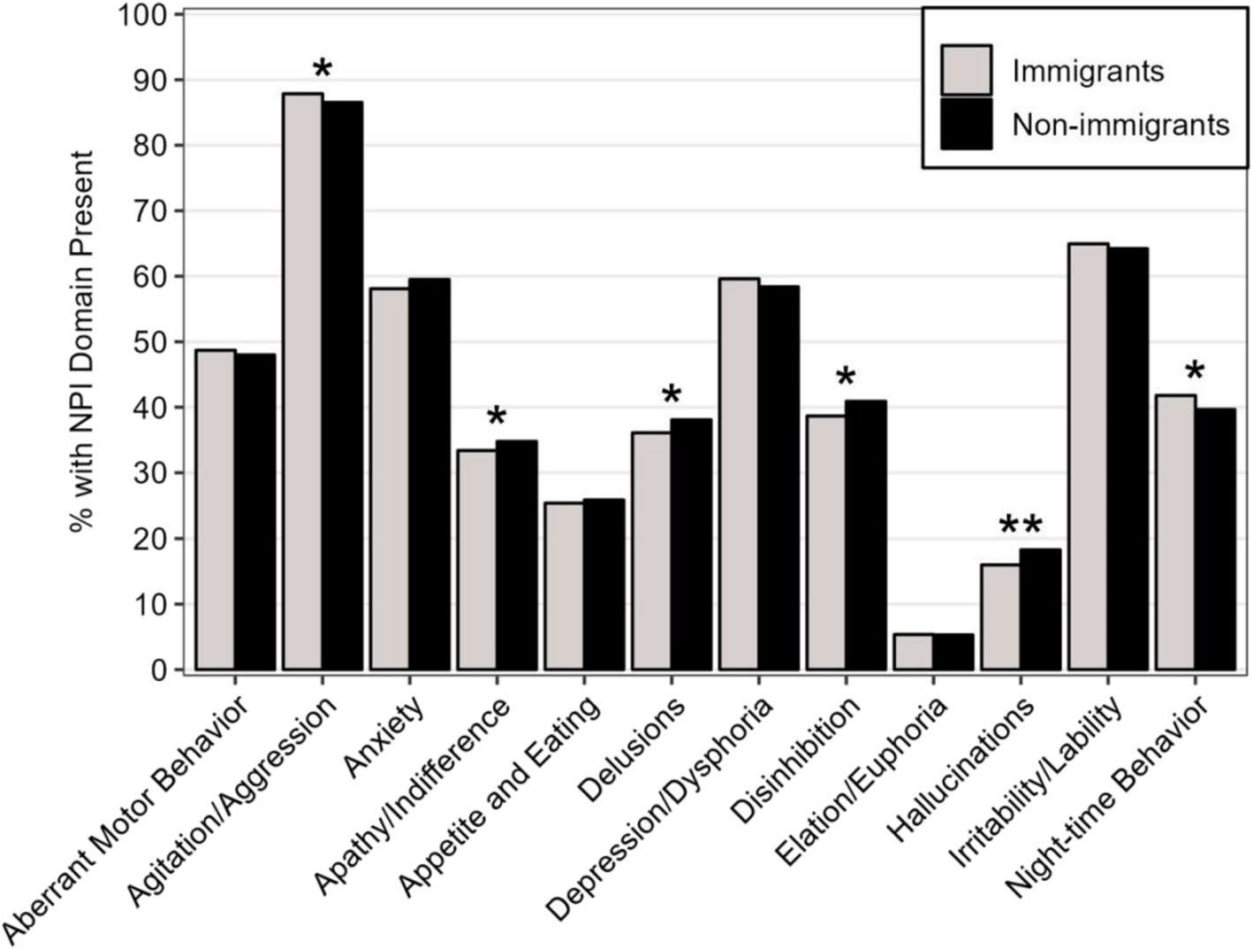
Prevelance of each NPI domain for immigrants and non-immigrants. NPI: neuropsychiatric inventory. Differences between the groups at the .05 level and .001 level are marked with * and **, respectively

There were no differences in the total NPI severity scores and the total number of NPI domains between immigrants and non-immigrants (Table 1). None of the NPI total scores had significant differences between English-speaking immigrants and non-immigrants. However, there was a significant difference in the total number of NPI domains (Cohen’s *d* = -0.06 [-0.09, - 0.02], *p* <.001) between non-English-speaking immigrants and non-immigrants (See Additional file 1). Individual NPI domain severity and prevalence scores are displayed in Table 2 and Fig. 1, respectively. The most frequently presenting NPS for immigrants and non-immigrant groups were agitation/aggression (87.9% and 86.5%), irritability/liability (65.0% and 64.2%), depression/dysphoria (59.6% and 58.4%), and anxiety (58.1% and 59.5%) (Fig. 1). However, significant differences were observed only for the prevalence of hallucinations (16.0% and 18.3%). In comparing non-English-speaking immigrants and non-immigrants, non-immigrants had a significantly higher prevalence of delusions, disinhibition, and hallucinations (See Additional file 2). There were no significant differences in individual NPI domain prevalence observed between English-speaking immigrants and non-immigrants (See Additional file 3). The prevalence of each domain for each group with the 95% confidence intervals is shown in Supplementary Table 2 (See Additional file 4).

**Table 2:** Individual NPI domain severity scores for non-immigrants, immigrants, NES immigrants, and ES immigrants

**Domain**	**Non-immigrants** **M(SD)**	**Immigrants** **M(SD)**	**NES immigrants** **M(SD)**	**ES immigrants** **M(SD)**
Aberrant Motor Behavior	1.0 (1.2)	1.1 (1.2)	1.0 (1.2)	1.1 (1.2)
Agitation/Aggression	1.9 (1.0)	1.9 (1.0)	1.9 (1.0)	2.0 (1.0)
Anxiety	1.3 (1.2)	1.3 (1.2)	1.2 (1.2)	1.3 (1.2)
Apathy/Indifference	0.7 (1.0)	0.7 (1.0)	0.7 (1.0)	0.7 (1.0)
Appetite and Eating	0.5 (0.9)	0.5 (0.9)	0.5 (0.9)	0.5 (0.9)
Delusions	0.8 (1.1)	0.7 (1.1)	0.7 (1.1)	0.8 (1.1)
Depression/Dysphoria	1.2 (1.1)	1.2 (1.1)	1.2 (1.1)	1.2 (1.1)
Disinhibition	0.9 (1.1)	0.8 (1.1)	0.8 (1.1)	0.9 (1.1)
Elation/Euphoria	0.1 (0.4)	0.1 (0.4)	0.1 (0.4)	0.1 (0.4)
Hallucinations	0.4 (0.8)	0.3 (0.8)	0.3 (0.7)	0.3 (0.8)
Irritability/Lability	1.4 (1.2)	1.4 (1.2)	1.4 (1.2)	1.5 (1.2)
Night-time Behavior	0.8 (1.1)	0.9 (1.1)	0.9 (1.1)	0.9 (1.1)

*NPI* Neuropsychiatric inventory, *NES* Non-English-speaking, *ES* English-speaking, *M* Mean, *SD* Standard deviation

A logistic regression, controlling for age and sex, examined the relationship between immigrant status and individual NPI domain severity scores (Table 3). There were significant effects for agitation/aggression and hallucinations for immigrant and non-immigrant groups. For every 1-point increase in agitation/aggression, it was 1.08 times more likely that a person was an immigrant. Conversely, for every 1-point increase in hallucinations, it was 1.07 times less likely that a person was an immigrant. In comparing non-English-speaking immigrants and non-immigrants, agitation/aggression, anxiety, depression/dysphoria, disinhibition, and hallucinations showed significant effects (Table 3).

**Table 3:** Logistic regression model predicting immigrant status from NPS severity, controlling for age and sex

	**Immigrants**					**NES immigrants**					**ES immigrants**				
**Term**	***B***	**SE**	***Z***	**OR [95% ** ***CI*** **]**	***p***	***B***	**SE**	***Z***	**OR [95% ** ***CI*** **]**	***p***	***B***	**SE**	***Z***	**OR [95% ** ***CI*** **]**	***p***
(intercept)	-2.27	0.18	-12.53	0.10 [0.07, 0.15]	<.001	-3.33	0.21	-15.68	0.04 [0.02, 0.05]	<.001	-2.15	0.26	-8.16	0.12 [0.07, 0.19]	<.001
Aberrant Motor Behavior	0.03	0.01	2.05	1.03 [1.00, 1.05]	.041	0.01	0.02	0.75	1.01 [0.98, 1.04]	.451	0.05	0.02	2.76	1.06 [1.02, 1.10]	.006
Agitation/Aggression	0.08	0.02	4.54	1.08 [1.05, 1.12]	<.001	0.09	0.02	4.37	1.09 [1.05, 1.14]	<.001	0.06	0.03	2.29	1.06 [1.01, 1.12]	.022
Anxiety	-0.03	0.01	-2.43	0.97 [0.94, 0.99]	.015	-0.06	0.02	-3.39	0.95 [0.92, 0.98]	<.001	0	0.02	0.23	1.00 [0.96, 1.05]	.814
Apathy/Indifference	-0.04	0.02	-2.78	0.96 [0.93, 0.99]	.005	-0.05	0.02	-2.6	0.95 [0.92, 0.99]	.009	-0.04	0.02	-1.58	0.96 [0.92, 1.01]	.114
Appetite and Eating	-0.02	0.02	-1.22	0.98 [0.95, 1.01]	.222	-0.03	0.02	-1.31	0.97 [0.93, 1.01]	.190	-0.01	0.03	-0.53	0.99 [0.94, 1.04]	.599
Delusions	-0.04	0.02	-2.36	0.96 [0.94, 0.99]	.018	-0.05	0.02	-2.9	0.95 [0.92, 0.98]	.004	-0.01	0.02	-0.29	0.99 [0.95, 1.04]	.773
Depression/Dysphoria	0.04	0.01	2.96	1.05 [1.01, 1.08]	.003	0.06	0.02	3.52	1.06 [1.03, 1.10]	<.001	0.01	0.02	0.64	1.01 [0.97, 1.06]	.523
Disinhibition	-0.05	0.01	-3.22	0.95 [0.93, 0.98]	.001	-0.07	0.02	-3.86	0.94 [0.91, 0.97]	<.001	-0.01	0.02	-0.5	0.99 [0.95, 1.03]	.615
Elation/Euphoria	0.03	0.04	0.8	1.03 [0.96, 1.11]	.425	0.01	0.04	0.32	1.01 [0.93, 1.10]	.752	0.05	0.05	0.93	1.05 [0.95, 1.16]	.350
Hallucinations	-0.07	0.02	-3.31	0.93 [0.90, 0.97]	<.001	-0.09	0.02	-3.67	0.91 [0.87, 0.96]	<.001	-0.03	0.03	-1.13	0.97 [0.91, 1.02]	.260
Irritability/Lability	0.02	0.01	1.33	1.02 [0.99, 1.05]	.185	0.01	0.02	0.75	1.01 [0.98, 1.05]	.454	0.03	0.02	1.43	1.03 [0.99, 1.08]	.152
Night-time Behavior	0.04	0.01	2.91	1.04 [1.01, 1.07]	.004	0.05	0.02	3.21	1.05 [1.02, 1.09]	.001	0.02	0.02	0.98	1.02 [0.98, 1.06]	.326
Age	0.02	0	8.78	1.02 [1.01, 1.02]	<.001	0.03	0	10.86	1.03 [1.02, 1.03]	<.001	0	0	1.02	1.00 [1.00, 1.01]	.309
Sex - Male	0.12	0.03	3.88	1.13 [1.06, 1.20]	<.001	0.13	0.04	3.72	1.14 [1.07, 1.23]	<.001	0.09	0.05	2.03	1.10 [1.00, 1.20]	.042

*NPS* Neuropsychiatric symptoms, *NES* Non-English-speaking, *ES* English-speaking,* B*: unstandardized coefficient, *SE* Standard error, *Z z* score for the Wald test, *OR* Odds ratio, *CI* Confidence interval; *p*: probability value. The reference group is non-immigrants

A linear regression model with immigrant status, age, and sex as predictors, was created using total NPI severity scores as the dependent variable. Across all the groups, immigrant status did not predict total NPI severity scores in the model when controlling for age and sex (See Additional file 5).

### Caregiver distress

There were no significant differences in total caregiver distress scores between all groups (Table 1). A linear regression model with immigrant status, age, and sex as predictors was created, using total caregiver distress scores as dependent variables. For all groups, immigrant status did not predict total caregiver distress when controlling for age and sex. However, age and sex had significant effects, with caregivers of older people and male referrals having lower distress scores across all comparison groups (See Additional file 6).

### Contributing factors to NPS

The prevalence of common contributing factors and two additional factors (language barriers and cultural considerations), which are directly related to our study objectives were compared across groups (Table 4). Only language barriers and cultural considerations accounted for significant differences between the two groups, with 6.5% and 2.2% higher prevalence, respectively for immigrants compared to non-immigrants. Loneliness/boredom, language barriers, and cultural considerations significantly contributed to NPS for non-English-speaking immigrants and non-immigrants comparison, with the largest significant differences being for language barriers (10.0%) and cultural considerations (3.4%). There were no differences in the prevalence of contributing factors between English-speaking immigrants and non-immigrants.

**Table 4:** Differences in the prevalence of commonly identified contributing factors to NPS for non-immigrants and immigrant groups

**Contributing factor**	**Non-immigrants** **%**	**Immigrants** **Difference [95% ** ***CI], p***	**NES immigrants** **Difference [95% ** ***CI], p***	**ES immigrants** **Difference [95% ** ***CI], p***
Pain	58.6%	1.5% [ 0.1, 2.9], *p* =.031	2.6% [ 1.0, 4.2], *p* =.001	-0.6% [-2.7, 1.5], *p* =.600
Carer approach	36.3%	-0.1% [-1.5, 1.2], *p* =.861	0.1% [-1.5, 1.6], *p* =.927	-0.5% [-2.6, 1.5], *p* =.609
Mood disorders	26.5%	-0.6% [-1.9, 0.6], *p* =.334	0.3% [-1.2, 1.7], *p* =.734	-2.3% [-4.1, -0.5], *p* =.015
Over/under stimulation	15.5%	0.1% [-0.9, 1.2], *p* =.816	-0.2% [-1.4, 0.9], *p* =.711	0.8% [-0.7, 2.4], *p* =.295
Loneliness/boredom	15.2%	1.4% [ 0.4, 2.5], *p* =.006	2.0% [ 0.8, 3.2], *p* <.001	0.4% [-1.2, 1.9], *p* =.652
Language barrier	0.2%	6.5% [ 6.0, 7.1], *p* <.001	10.0% [ 9.1, 10.8], *p* <.001	-0.0% [-0.3, 0.2], *p* =.861
Cultural considerations	0.5%	2.2% [ 1.8, 2.6], *p* <.001	3.4% [ 2.8, 3.9], *p* <.001	-0.1% [-0.3, -0.0], *p* =.190

*NPS* Neuropsychiatric symptoms, *NES* Non-English-speaking, *ES* English-speaking, *CI* Confidence interval, *p*: probability

## Discussion

This study compared the clinical and demographic characteristics, prevalence and severity of NPS, and associated caregiver distress for immigrants and non-immigrants living with dementia in RACHs referred to an Australian national dementia support service (DSA). It is important to note that our sample consisted of referrals who exhibited NPS at levels requiring specialist support. We analysed data collected from the first referral to DSA and as such, our findings reflect the level of symptoms requiring support and not the impact of support for NPS. With 24% of referrals from non-English-speaking countries, our sample was comparable to the national record that estimates 23% of Australians aged 65 and over were born in non-English-speaking countries [1]. Additionally, Alzheimer’s disease, the most common form of dementia worldwide [47] was the most represented dementia subtype in both immigrant and non-immigrant groups. We found differences between immigrants and non-immigrants in age and dementia subtype, but not sex, partially supporting our first hypothesis. Our second hypothesis was also partially supported as we observed differences in the individual NPI domain severity scores between the two groups, but not in total severity scores. Our third hypothesis was not supported as there were no significant differences in caregiver distress between immigrants and non-immigrants living with dementia. However, there were differences in the prevalence of factors contributing to NPS between the two groups, thus confirming our fourth hypothesis.

### Neuropsychiatric symptoms (Hypothesis 2)

Our findings indicated a significantly lower rate of hallucinations for immigrants and non-English-speaking immigrants compared to non-immigrants. Additionally, non-English-speaking immigrants were significantly lower on delusions and disinhibition than non-immigrants. The literature reports different types of NPS and higher frequencies of some NPS for ethnic minorities compared with their counterparts or native populations. Salazar and colleagues [11] compared NPI scores for ethnic groups living with Alzheimer’s disease in the community (*n* = 975) reporting a higher frequency of hallucinations, night-time behaviour, and elation for people from Hispanic backgrounds. However, Wilson and colleagues [48] reported no differences in NPS presentation for Latino older adults and non-Latino older adults living with dementia in Brazil, reasoning that NPS are driven primarily by brain structure and functional changes than by sociodemographic characteristics. However, the findings of these studies are different to our study findings likely reflecting the differences in the study methodology (e.g., study design) and sample characteristics (e.g., sample size).

Our study found no differences in total NPI severity scores between immigrants and non-immigrants. This is partially due to differences in the direction of individual domains cancelling out the effect in the total severity scores, where immigrants were more likely to be higher on agitation/aggression but lower on hallucinations, compared to non-immigrants. This also applies to the comparison for non-English-speaking immigrants, which were more likely to be higher on agitation/aggression and depression/dysphoria, but lower on anxiety, disinhibition, and hallucinations. The higher severity of agitation/aggression is likely driven by communication difficulties as there was no difference for the English-speaking immigrant groups comparison. Cognitive decline can impair both the ability to express and comprehend spoken language and people living with dementia who have English as their additional language may lose their ability to communicate in English and subsequently use their first language as the primary language of communication [7, 8]. A study comparing verbal communication and psychiatric medication use by Greek and Italian residents with dementia in ethno-specific and mainstream RACHs in Australia found that communication difficulties were linked with higher rates of psychiatric prescriptions, ostensibly for managing NPS such as agitation and aggression [49]. There were no significant differences in NPI prevalence and total NPI severity between English-speaking immigrants and non-immigrants highlighting the impact of language and culture in NPS presentation in people living with dementia. Unlike non-English-speaking immigrants, English-speaking immigrants may not have experienced communication and/or cultural difficulties with their formal caregivers.

### Caregiver distress (Hypothesis 3)

There was no difference in caregiver distress between caregivers of immigrants and non-immigrants living with dementia. This may be because our sample comprised of referrals for NPS support from RACHs and as such similar levels of distress have been reported by the caregivers across the groups. Further, formal caregivers working in RACHs may have had training on managing NPS and regularly supported such symptoms. Current literature suggests longer durations of care [50, 51] and more caregiving activities [50] are linked to increased informal caregiver distress and burden.

### Contributing factors to NPS (Hypothesis 4)

Pain was the top contributing factor for NPS for both immigrant and non-immigrant groups, echoing previous findings that pain is commonly associated with NPS, particularly agitation/aggression [27, 52]. Given that pain is a frequently occurring contributing factor to NPS, future studies should focus on examining the association between pain and NPS. We found a higher rate of language barriers and cultural considerations for non-English-speaking immigrants compared to non-immigrants. Such findings support what is already known about the impact of culture on the patterns of NPS presentations for people living with dementia and where there is a lack of cultural considerations and communication options available in RACHs this may exacerbate agitation [53, 54]. For this study pain was assessed using PainChek^®^, an observational pain assessment instrument that alleviates the need for verbal communication and self-reporting of pain. While such tools are important for people who have lost language expression, lack of such options can be difficult for carers in understanding the expressions of pain by residents from immigrant backgrounds. Data from the Australian Royal Commission into Aged Care Quality and Safety provides examples of how care workers' inability to recognize pain expression of residents from immigrant backgrounds who have lost their English language led to poor care outcomes [55].

### Strengths and limitations

This is one of the largest population-based studies and to the best of our knowledge, the first in Australia, to comparatively describe the clinical, demographic, and NPS characteristics of immigrants and non-immigrants living with dementia referred to an Australian dementia behaviour support service. The strengths of this study include the use of a large representative sample of people living with dementia aged 65 years and above, strengthening the generalisability of our findings to older people living with dementia who received external support for NPS.

This study has some limitations that need to be noted. This was a retrospective observational cross-sectional study and bound by the limitations of observational studies such as the inability to attribute causation [56]. Our findings may be limited to participants aged 65 years and above and living in RACHs, although, there are lessons for community/home care that are relevant. Constrained by existing data variables, we could not investigate the potential confounding effects of racial and ethnic variations, medical conditions, medication profiles, and other covariates such as levels of cognitive function or activities of daily living. We determined immigrant status by documented country of birth, and we did not have access to the duration of stay in Australia (e.g., long-term immigrants or short-term immigrants) or English language proficiency; as such, we cannot be certain that people coming from non-English-speaking countries were speaking English as a first language or had developed and/or lost English as their second language. In general, contributing factors to NPS are complex and their identification is complicated by the (co-)presence of many behaviours often having numerous causes [57]. These factors are of various types including biological, psychological, social and/or environmental factors [25, 57]. Therefore, identifying any factor requires a robust and comprehensive assessment process and obtaining information through multiple sources including standardised and validated instruments, observation, interview, and review of clinical notes and social history. Due to the complexity of contributing factors and their assessment, their identification may be subject to bias and should be interpreted within this context. However, the risk of such bias is mitigated by the extensive clinical expertise and experience of DSA consultants. We have also not performed an adjustment for multiple testing due to the exploratory nature of our study. However, we have interpreted the significance of our findings in relation to a stricter alpha value of <.001 rather than <.05. Further, due to the exploratory nature of the hypotheses, our findings need to be replicated in future studies to confirm any conclusions.

## Conclusion

Our findings reveal a mixed picture of NPS between immigrants and non-immigrants living with dementia that may give policymakers and service providers a better picture of the potential differences in demographic characteristics, prevalence and severity of NPS, and factors contributing to NPS. This may be useful for future service planning, in particular, but not limited to RACHs. Our findings call for increased awareness and education on the impact of culture and language on NPS for people receiving residential care. Future studies where variables such as length of stay in Australia and English language proficiency can be determined are needed as are those with diverse study designs (e.g., longitudinal studies) to learn more about NPS presentations for different immigrant groups (e.g., first-and second-generation immigrants). Studies investigating the association between NPS and pain intensity in people living with dementia, with different migration histories, will also contribute to the appropriate management of NPS.

### Supplementary Information

**Additional file 1: Supplementary Table 1. **Effect sizes, 95% CI and p-values for the descriptive characteristics of NES and ES immigrants.

** Additional file 2: Supplementary Figure 1. **Prevalence of each NPI domain for NES immigrants and non-immigrants. NES: non-English-speaking; NPI: neuropsychiatric inventory. Differences between the groups at the .05 level and .001 level are marked with * and **, respectively.

** Additional file 3: Supplementary Figure 2.** Prevalence of each NPI domain for ES immigrants and non-immigrants. NPI: neuropsychiatric inventory; ES: English-speaking. Differences between the groups at the .05 level are marked with *.

** Additional file 4: Supplementary Table 2.** Prevalence of each NPS domain for each group with the 95% confidence intervals. 

** Additional file 5: Supplementary Table 3.** Linear regression model predicting total NPI severity scores from immigrant status controlling for age and sex. 

** Additional file 6: Supplementary Table 4.** Linear regression model predicting total caregiver distress scores from immigrant status controlling for age and sex.

## Data Availability

Restrictions apply to the availability of data generated or analysed during this study to preserve data privacy. The corresponding author will, on request, provide details on the restrictions and any conditions under which access to some data may be provided.

## Abbreviations

DBMAS: Dementia Management Advisory Service
DSA: Dementia Support Australia
NPI: Neuropsychiatric Inventory
NPI-NH: Neuropsychiatry Inventory Nursing Home
NPI-Q: Neuropsychiatry Inventory Questionnaire
NPS: Neuropsychiatric symptoms
RACH(s): Residential Aged Care Home(s)
SBRT: Severe Behaviour Response Team
