# Supplementary material for: Comparing clinico-demographics and neuropsychiatric symptoms for immigrant and non-immigrant aged care residents living with dementia: a retrospective cross-sectional study from an Australian dementia-specific support service
Source: BMC Geriatr. 2023 Nov 10;23:729. doi: 10.1186/s12877-023-04447-3 (PMC10636936; doi:10.1186/s12877-023-04447-3)
Supplement: Supplementary file 1 — Additional file 1: Supplementary Table 1. Effect sizes, 95% CI and p-values for the descriptive characteristics of NES and ES immigrants. [file 12877_2023_4447_MOESM1_ESM.docx]

**Supplementary Table 1.** Effect sizes, 95% *CI* and *p*-values for the descriptive characteristics of NES and ES immigrants.

| **Group** | **NES immigrants** | | **ES immigrants** | |
| --- | --- | --- | --- | --- |
|  | **Effect size [95% *CI*]** | ***p*** | **Effect size [95% *CI*]** | ***p*** |
| Age | 0.18 [ 0.15, 0.21]* | < .001 | 0.00 [-0.04, 0.04]* | .851 |
| Sex | 0.02 [ 0.00, 0.03]** | .004 | 0.01 [ 0.00, 0.02]** | .135 |
| Dementia subtype | 0.05 [ 0.03, 0.06]** | < .001 | 0.04 [ 0.01, 0.05]** | < .001 |
| NPI Totals |  |  |  |  |
| Severity | -0.04 [-0.08, -0.01]* | .012 | 0.07 [ 0.02, 0.11]* | .002 |
| Caregiver distress | -0.03 [-0.06, 0.01]* | .113 | 0.05 [ 0.00, 0.09]* | .030 |
| Number of Domains | -0.06 [-0.09, -0.02]* | < .001 | 0.06 [ 0.02, 0.11]* | .005 |
| Pain Present | 0.02 [ 0.00, 0.04]** | .064 | 0.02 [ 0.00, 0.04]** | .044 |

*Cohen’s *d* effect size; **Cramer’s *V* effect size; NES: non-English-speaking; NA: not applicable; NPI: neuropsychiatric inventory; *CI*: confidence interval; *p*: probability value.
