# Supplementary material for: Comparing clinico-demographics and neuropsychiatric symptoms for immigrant and non-immigrant aged care residents living with dementia: a retrospective cross-sectional study from an Australian dementia-specific support service
Source: BMC Geriatr. 2023 Nov 10;23:729. doi: 10.1186/s12877-023-04447-3 (PMC10636936; doi:10.1186/s12877-023-04447-3)
Supplement: Supplementary file 2 — Additional file 2: Supplementary Figure 1. Prevalence of each NPI domain for NES immigrants and non-immigrants. NES: non-English-speaking; NPI: neuropsychiatric inventory. Differences between the groups at the .05 level and .001 level are marked with * and **, respectively. [file 12877_2023_4447_MOESM2_ESM.pdf]

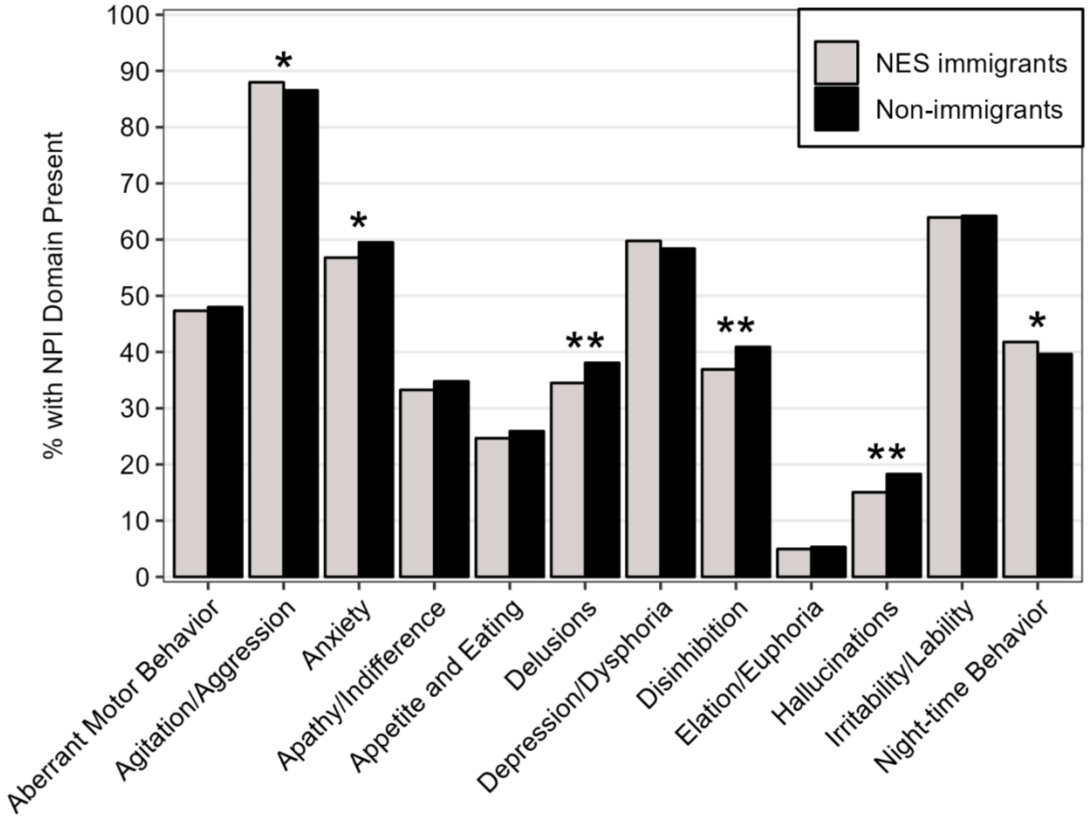

Supplementary Figure 1. Prevalence of each NPI domain for NES immigrants and non-immigrants.

NES: non-English-speaking; NPI: neuropsychiatric inventory. Differences between the groups at the .05 level and .001 level are marked with * and **, respectively.
