# Supplementary material for: Comparing clinico-demographics and neuropsychiatric symptoms for immigrant and non-immigrant aged care residents living with dementia: a retrospective cross-sectional study from an Australian dementia-specific support service
Source: BMC Geriatr. 2023 Nov 10;23:729. doi: 10.1186/s12877-023-04447-3 (PMC10636936; doi:10.1186/s12877-023-04447-3)
Supplement: Supplementary file 3 — Additional file 3: Supplementary Figure 2. Prevalence of each NPI domain for ES immigrants and non-immigrants. NPI: neuropsychiatric inventory; ES: English-speaking. Differences between the groups at the .05 level are marked with *. [file 12877_2023_4447_MOESM3_ESM.pdf]

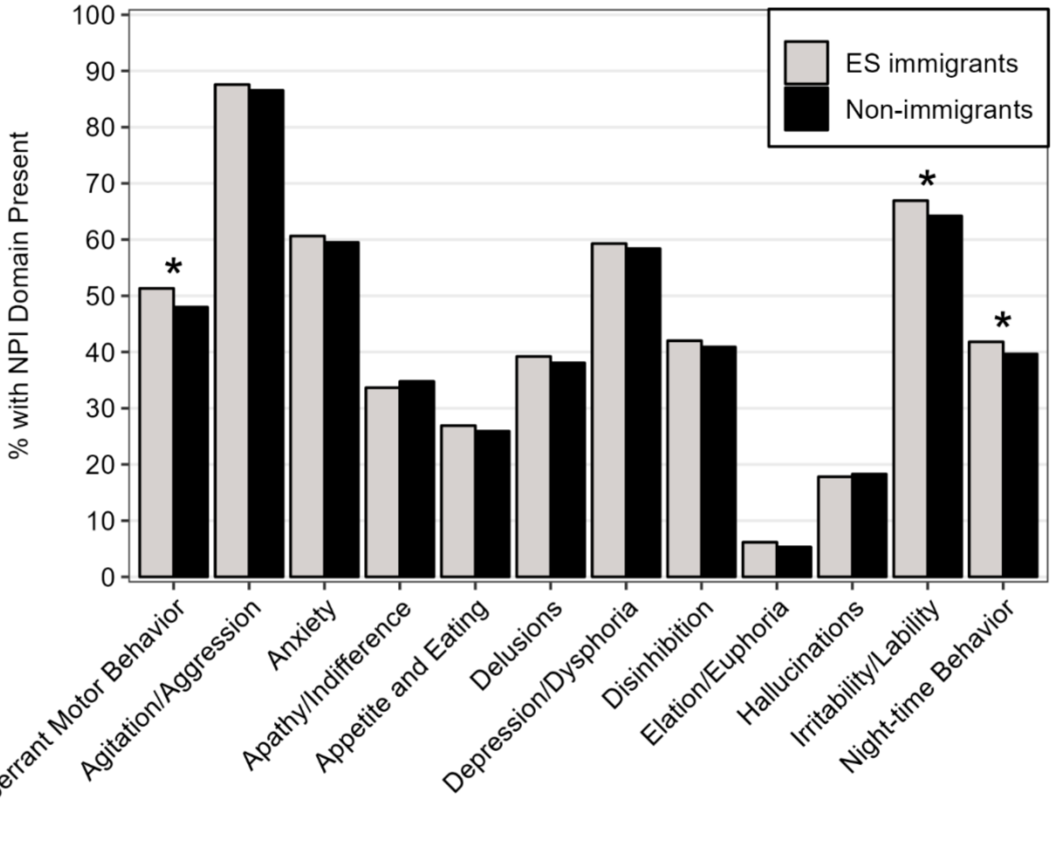

Supplementary Figure 2. Prevalence of each NPI domain for ES immigrants and non-immigrants.

NPI: neuropsychiatric inventory; ES: English-speaking. Differences between the groups at the .05 level are marked with *.
