# Supplementary material for: Comparing clinico-demographics and neuropsychiatric symptoms for immigrant and non-immigrant aged care residents living with dementia: a retrospective cross-sectional study from an Australian dementia-specific support service
Source: BMC Geriatr. 2023 Nov 10;23:729. doi: 10.1186/s12877-023-04447-3 (PMC10636936; doi:10.1186/s12877-023-04447-3)
Supplement: Supplementary file 4 — Additional file 4: Supplementary Table 2. Prevalence of each NPS domain for each group with the 95% confidence intervals. [file 12877_2023_4447_MOESM4_ESM.docx]

**Supplementary Table 2.** Prevalence of each NPS domain for each group with the 95% confidence intervals.

| **Domain** | **Non-immigrants** | **NES Immigrants** | **ES Immigrants** | **Immigrants** |
| --- | --- | --- | --- | --- |
| Aberrant Motor Behavior | 48.0% [47.6, 48.4] | 47.3% [46.6, 48.0] | 51.3% [50.3, 52.3] | 48.7% [48.1, 49.3] |
| Agitation/Aggression | 86.5% [86.2, 86.8] | 88.0% [87.5, 88.5] | 87.6% [86.9, 88.2] | 87.9% [87.5, 88.2] |
| Anxiety | 59.5% [59.1, 59.9] | 56.8% [56.1, 57.5] | 60.6% [59.7, 61.6] | 58.1% [57.5, 58.7] |
| Apathy/Indifference | 34.8% [34.4, 35.2] | 33.3% [32.6, 33.9] | 33.6% [32.7, 34.6] | 33.4% [32.8, 33.9] |
| Appetite and Eating | 25.9% [25.5, 26.3] | 24.7% [24.0, 25.3] | 26.9% [26.0, 27.8] | 25.4% [24.9, 25.9] |
| Delusions | 38.1% [37.7, 38.5] | 34.5% [33.8, 35.2] | 39.2% [38.2, 40.2] | 36.1% [35.5, 36.6] |
| Depression/Dysphoria | 58.4% [58.0, 58.8] | 59.8% [59.1, 60.5] | 59.3% [58.3, 60.3] | 59.6% [59.0, 60.2] |
| Disinhibition | 40.9% [40.5, 41.3] | 36.9% [36.2, 37.6] | 42.0% [41.0, 43.0] | 38.7% [38.1, 39.2] |
| Elation/Euphoria | 5.3% [5.1, 5.5] | 4.9% [4.6, 5.3] | 6.2% [5.7, 6.6] | 5.4% [5.1, 5.6] |
| Hallucinations | 18.3% [17.9, 18.6] | 15.1% [14.5, 15.6] | 17.8% [17.0, 18.6] | 16.0% [15.6, 16.4] |
| Irritability/Lability | 64.2% [63.8, 64.6] | 63.9% [63.2, 64.6] | 66.9% [66.0, 67.9] | 65.0% [64.4, 65.5] |
| Night-time Behavior | 39.6% [39.2, 40.1] | 41.8% [41.1, 42.5] | 41.8% [40.8, 42.8] | 41.8% [41.2, 42.4] |

NPS: neuropsychiatric symptoms; NES: non-English-speaking; ES: English-speaking.
