# Supplementary material for: Comparing clinico-demographics and neuropsychiatric symptoms for immigrant and non-immigrant aged care residents living with dementia: a retrospective cross-sectional study from an Australian dementia-specific support service
Source: BMC Geriatr. 2023 Nov 10;23:729. doi: 10.1186/s12877-023-04447-3 (PMC10636936; doi:10.1186/s12877-023-04447-3)
Supplement: Supplementary file 5 — Additional file 5: Supplementary Table 3. Linear regression model predicting total NPI severity scores from immigrant status controlling for age and sex. [file 12877_2023_4447_MOESM5_ESM.docx]

Supplementary Table 3. Linear regression model predicting total NPI severity scores from immigrant status controlling for age and sex.

| Term | *B* [95% *CI*] | SE | *t* | *p* |
| --- | --- | --- | --- | --- |
| (Intercept) | 14.33 [ 13.39, 15.27] | 0.48 | 29.87 | <.001 |
| **Immigrant - Yes** | 0.03 [-0.14 , 0.20] | 0.09 | 0.33 | .740 |
| Age | -0.04 [-0.05 , -0.03] | 0.01 | -6.76 | <.001 |
| Sex - Male | -0.83 [-0.99 , -0.66] | 0.08 | -9.86 | <.001 |
|  |  |  |  |  |
| (Intercept) | 14.24 [ 13.24, 15.24] | 0.51 | 27.87 | <.001 |
| **NES immigrant - Yes** | -0.17 [-0.37 , 0.02] | 0.10 | -1.72 | .085 |
| Age | -0.04 [-0.05 , -0.03] | 0.01 | -6.18 | <.001 |
| Sex - Male | -0.80 [-0.98 , -0.63] | 0.09 | -8.92 | <.001 |
|  |  |  |  |  |
| (Intercept) | 13.96 [ 12.90, 15.03] | 0.54 | 25.72 | <.001 |
| **ES immigrant - Yes** | 0.41 [ 0.16 , 0.66] | 0.13 | 3.18 | .001 |
| Age | -0.03 [-0.05 , -0.02] | 0.01 | -5.27 | <.001 |
| Sex - Male | -0.84 [-1.03 , -0.65] | 0.10 | -8.65 | <.001 |

NPI: neuropsychiatric inventory; *CI:* confidence interval; *B*: unstandardized coefficient; SE: standard error; *t*: t score value; *p*: probability value; NES: non-English-speaking; ES: English-speaking. The reference group is non-immigrants.
