# Supplementary material for: Comparing clinico-demographics and neuropsychiatric symptoms for immigrant and non-immigrant aged care residents living with dementia: a retrospective cross-sectional study from an Australian dementia-specific support service
Source: BMC Geriatr. 2023 Nov 10;23:729. doi: 10.1186/s12877-023-04447-3 (PMC10636936; doi:10.1186/s12877-023-04447-3)
Supplement: Supplementary file 6 — Additional file 6: Supplementary Table 4. Linear regression model predicting total caregiver distress scores from immigrant status controlling for age and sex. [file 12877_2023_4447_MOESM6_ESM.docx]

Supplementary Table 4. Linear regression model predicting total caregiver distress scores from immigrant status controlling for age and sex.

| Term | *B* [95% *CI*] | SE | *t* | *p* |
| --- | --- | --- | --- | --- |
| (Intercept) | 19.07 [ 17.68, 20.45] | 0.71 | 26.99 | <.001 |
| **Immigrant - Yes** | 0.06 [-0.19 , 0.31] | 0.13 | 0.46 | .648 |
| Age | -0.05 [-0.07 , -0.03] | 0.01 | -5.96 | <.001 |
| Sex - Male | -0.86 [-1.10 , -0.62] | 0.12 | -6.96 | <.001 |
|  |  |  |  |  |
| (Intercept) | 19.16 [ 17.69, 20.64] | 0.75 | 25.46 | <.001 |
| **NES immigrant - Yes** | -0.13 [-0.42 , 0.16] | 0.15 | -0.89 | .372 |
| Age | -0.05 [-0.07 , -0.03] | 0.01 | -5.72 | <.001 |
| Sex - Male | -0.85 [-1.11 , -0.59] | 0.13 | -6.44 | <.001 |
|  |  |  |  |  |
| (Intercept) | 18.57 [ 17.01, 20.13] | 0.80 | 23.33 | <.001 |
| **ES immigrant - Yes** | 0.42 [ 0.05 , 0.79] | 0.19 | 2.24 | .025 |
| Age | -0.04 [-0.06 , -0.03] | 0.01 | -4.67 | <.001 |
| Sex - Male | -0.83 [-1.11 , -0.55] | 0.14 | -5.86 | <.001 |

NPI: neuropsychiatric inventory; *CI*: confidence interval; *B*: unstandardized coefficient: SE: standard error; *t*: t score value; *p*: probability value; NES: non-English-speaking; ES: English-speaking. The reference group is non-immigrants.
